# Eradication of Helicobacter pylori in the Primary Prevention of Gastric Cancer: A Systematic Review and Meta-Analysis

**DOI:** 10.7759/cureus.102401

**Published:** 2026-01-27

**Authors:** Felipe A. Muñoz-Rossi, Pablo Guillermo Hernandez-Almonacid, Ximena Marin-Quintero, Reina M Macero, Patricia León-León, Alba Del Pilar Rodriguez-Cortes, Sandra Milena Pulido León, Susana Ramírez Arcila, Gina Paola Ricardo Ossio

**Affiliations:** 1 Department of Internal Medicine, National University of Colombia, Bogotá, COL; 2 Methodology of Health Sciences Research, International University of La Rioja, Bogotá, COL; 3 Department of Pathology and Laboratory Medicine, National University of Colombia, Bogotá, COL; 4 Department of Biochemistry, Universidad de Cuenca, Cuenca, ECU; 5 Department of Human Reproduction and Infertility, Human Reproduction Institute of Guayaquil, Guayaquil, ECU; 6 Methodology of Health Sciences Research, University of Guayaquil, Faculty of Medical Sciences, Guayaquil, ECU; 7 Faculty of Health Sciences/Nursing Program, University of Pamplona, Cúcuta, COL; 8 Department of Medicine, San Martín University Foundation, Sabaneta, COL; 9 Department of Medical Affairs, Universidad del Rosario, Bogotá, COL; 10 Department of Clinical Research, Universidad Metropolitana de Barranquilla, Barranquilla, COL

**Keywords:** gastric cancer (gc), gastric cancer prevention, helicobacter pylori treatment, helicobacter pylori, primary cancer prevention

## Abstract

*Helicobacter* infection is the main modifiable risk factor for gastric cancer, especially in regions with intermediate to high incidence. Although *Helicobacter pylori* eradication has been shown to have a potential preventive effect, its magnitude remains a subject of debate due to methodological differences between clinical trials. We conducted a systematic review and meta-analysis, prospectively registered in PROSPERO (CRD420251181762), to evaluate the effect of *H. pylori* eradication on the incidence of gastric cancer in asymptomatic adults. A systematic search of major databases was conducted through November 2025, including randomized clinical trials that compared eradication therapy with control interventions. In most of the included studies, eradication therapy was consistently associated with a reduction in gastric cancer incidence, with moderate variability between trials. Sensitivity analyses supported the robustness of this association, while no clear effect on gastric cancer-specific mortality was observed. Our findings suggest that *H. pylori* eradication represents an effective strategy for the primary prevention of gastric cancer in populations at intermediate to high risk of developing the disease.

## Introduction and background

*Helicobacter pylori* infection is a very common disease worldwide. Although its incidence is declining in developing countries due to improved health policies and widespread use of antibiotics, it remains above 50% in most countries [1]. More than two-thirds of the population is infected with *H. pylori*, which can be asymptomatic, showing an unpredictable spectrum ranging from chronic gastritis, present in all infected individuals, to potentially serious lesions such as gastric cancer.

In Colombia, at least 80% of the population is infected with *H. pylori*, and as a result, gastric cancer is the infectious disease with the highest mortality rate [2]. The likelihood of contracting *H. pylori* infection is influenced by socioeconomic status and living conditions during childhood [3]. Various factors such as overcrowding, number of siblings, bed sharing, and lack of drinking water have been linked to an increased risk of *H. pylori* infection. It is unclear how it spreads, but evidence suggests that family members tend to be affected, suggesting the importance of exposure through fecal-oral or oral-oral contact [3]. Humans are the main reservoir of infection [4]; however, *H. pylori* has been identified in sheep's milk and stomach secretions [5]. Water supply systems in underdeveloped countries could serve as a source of bacteria in endemic areas [2]. The isolation of *H. pylori* from dental caries increases the risk of exposure and oral-oral transmission [6].

*H. pylori* has been identified as a carcinogenic agent based on extensive epidemiological and clinical evidence linking chronic infection to gastric malignancy. Meta-analytic data suggest that eradication therapy is associated with a reduced risk of developing gastric cancer when compared with no eradication treatment [7]. The probability of reinfection in adults is low; recurrence is more likely after partial suppression due to incomplete treatment or insufficient eradication of *H. pylori* [4]. *H. pylori* is not harmless; it always causes chronic gastritis and has the potential to develop into serious diseases. Therefore, it is essential to offer treatment to all infected individuals [8].

As a result of the high resistance of *H. pylori* due to the indiscriminate use of antibiotics associated with ineffective treatment regimens and failure to consider the local epidemiology of resistance in the region, it is recommended that treatment regimens used for the eradication of *H. pylori* have an intention-to-treat (ITT) efficacy of 90% [9]. Eradicating *H. pylori* interrupts various inflammatory mechanisms involved in gastric cancer, the severity of which varies among individuals due to bacterial virulence, environmental and genetic factors, and gastric microbial dysbiosis that predisposes to changes in the gastric mucosa [10].

It is believed that gastric cancer develops in the context of predominantly corpus gastritis, atrophy, and a profound loss of acid secretory capacity that precedes cancer by decades. This microenvironment, which is achlorhydric and inflamed, is made worse by a microbiota that is both pro-inflammatory and genotoxic, which causes the carcinogenic process to begin even after *H. pylori* has been eradicated [11].

Therefore, past research and meta-analyses show that eliminating *H. pylori* may lower the number of people who develop stomach cancer as well as the death rate associated with the disease. Because of this, the elimination of *H. pylori* is a fundamental method in primary prevention. This eliminates the principal carcinogen in stomach cancer before the mucosa undergoes alterations that are irreversible.

The objective of this meta-analysis is to synthesize the available and updated evidence on the real effect of *H. pylori* eradication on the incidence of gastric cancer, given that the results may guide beneficial actions in populations with a high incidence of gastric cancer and *H. pylori*.

## Review

Materials and methods

A systematic review with meta-analysis was performed to quantify the association between *H. pylori* eradication and the risk of gastric cancer in asymptomatic adult populations. The review methodology was developed in line with internationally accepted standards for intervention reviews, following guidance from the Cochrane Collaboration, and the reporting adhered to the Preferred Reporting Items for Systematic Reviews and Meta-Analyses (PRISMA) 2020 statement. The study protocol was prospectively registered in PROSPERO (CRD420251181762), ensuring methodological transparency and reproducibility.

Search Strategy

The systematic search was conducted until November 2025 in the MEDLINE/PubMed and Web of Science databases. Publications in English and Spanish were included, with no restriction on the year of publication.

A sensitive search strategy was used, combining controlled terms (Medical Subject Headings (MeSH)) and free-text terms related to *H. pylori* infection, eradication therapies, and gastric cancer, using Boolean operators. The complete search strategy and databases consulted are summarized in Table 1. Additionally, ClinicalTrials.gov was searched to identify ongoing or unpublished trials, and reference lists of the included studies were manually reviewed. A complementary snowball search was also performed.

**Table 1:** Search strategy for study identification This table summarizes the electronic search strategy used to identify eligible randomized clinical trials evaluating the effect of *Helicobacter pylori* eradication on gastric cancer incidence. The databases searched included MEDLINE (PubMed) and Web of Science. For each database, the table details the combination of MeSH terms and free-text keywords applied, the languages considered, the study design of interest, and the date of the last search (November 2025). MeSH, Medical Subject Headings

Database	Search terms (MeSH and free-text)	Language	Study design	Last search
MEDLINE (PubMed)	(“*Helicobacter pylori*” OR “*H. pylori* infection”) AND (“eradication therapy” OR “triple therapy” OR “quadruple therapy”) AND (“stomach neoplasms” OR “gastric cancer”) AND (“randomized controlled trial”)	English, Spanish	Randomized clinical trials	November 2025
Web of Science	(“*Helicobacter pylori*” OR “*H. pylori* infection”) AND (“eradication therapy” OR “triple therapy” OR “quadruple therapy”) AND (“gastric cancer” OR “stomach neoplasms”) AND randomized	English, Spanish	Randomized clinical trials	November 2025

Inclusion and Exclusion Criteria

Randomized clinical trials evaluating *H. pylori* eradication therapy in asymptomatic adults with confirmed infection were considered eligible. Studies were required to include a comparator group without intent to eradicate *H. pylori* and to report gastric cancer incidence or mortality outcomes with a minimum follow-up of three years. Trials enrolling patients with previous gastric cancer, advanced neoplastic lesions, or incomplete outcome data were excluded. The eligibility criteria and study framework are detailed in Table 2.

**Table 2:** PICO framework of the research question This table presents the PICO framework used to formulate the research question and define the eligibility criteria for study inclusion. It outlines the characteristics of the population, intervention, comparator, and outcomes of interest, as well as the study design considered for this systematic review and meta-analysis evaluating the effect of *Helicobacter pylori* eradication on gastric cancer incidence.

Component	Description
Population (P)	Asymptomatic adults with *Helicobacter pylori* infection confirmed by breath test, fecal antigen, or gastric biopsy
Intervention (I)	*H. pylori* eradication therapy (triple or quadruple regimens)p
Comparator (C)	Placebo or management without intent to eradicate *H. pylori*
Outcomes (O)	Primary: incidence of gastric cancer. Secondary: gastric cancer–specific mortality and reported effect measures (RR, OR, HR).
Study design	Randomized clinical trials

The selection of clinical trials was performed independently by the researchers, using the Rayyan platform, through title/abstract screening and full-text evaluation. Disagreements were resolved by consensus among the researchers.

Methodological Quality and Certainty of Evidence

The risk of bias in the included studies was assessed independently using the Cochrane Risk of Bias 2.0 (RoB 2.0) tool, considering the domains of random sequence generation, allocation concealment, blinding, incomplete outcome data, and selective reporting. Publication bias was explored using funnel plots and, when appropriate, adjustment methods. The overall certainty of the evidence was assessed using the GRADE methodology, considering risk of bias, inconsistency, indirectness, imprecision, and possible publication bias.

Data Extraction

Data extraction was performed by the same reviewers using a standardized, pre-piloted form. Information was collected on the author, year, and country of publication; study design; population characteristics; diagnostic method for *H. pylori*; eradication treatment regimen and duration; comparator; number of participants per group; follow-up time; number of incident cases of gastric cancer; associated mortality; and reported effect measures. When necessary, the corresponding authors were contacted to obtain additional information or clarify missing data.

Statistical Analysis

Dichotomous outcomes were summarized using relative risk (RR) with 95% confidence intervals. Statistical heterogeneity was assessed using chi-squared and I² statistics, considering values ≥50% as indicative of moderate to high heterogeneity. Depending on the degree of heterogeneity, fixed-effect or random-effect models were used. When significant unexplained heterogeneity was identified, the results were presented individually by study along with their respective confidence intervals. Statistical analyses were performed using R software (version 2025.09.2, R Core Team, R Foundation for Statistical Computing, Vienna, Austria), employing the meta and metafor packages (Wolfgang Viechtbauer, Maastricht University, Maastricht, Netherlands).

Results

**Figure 1:**
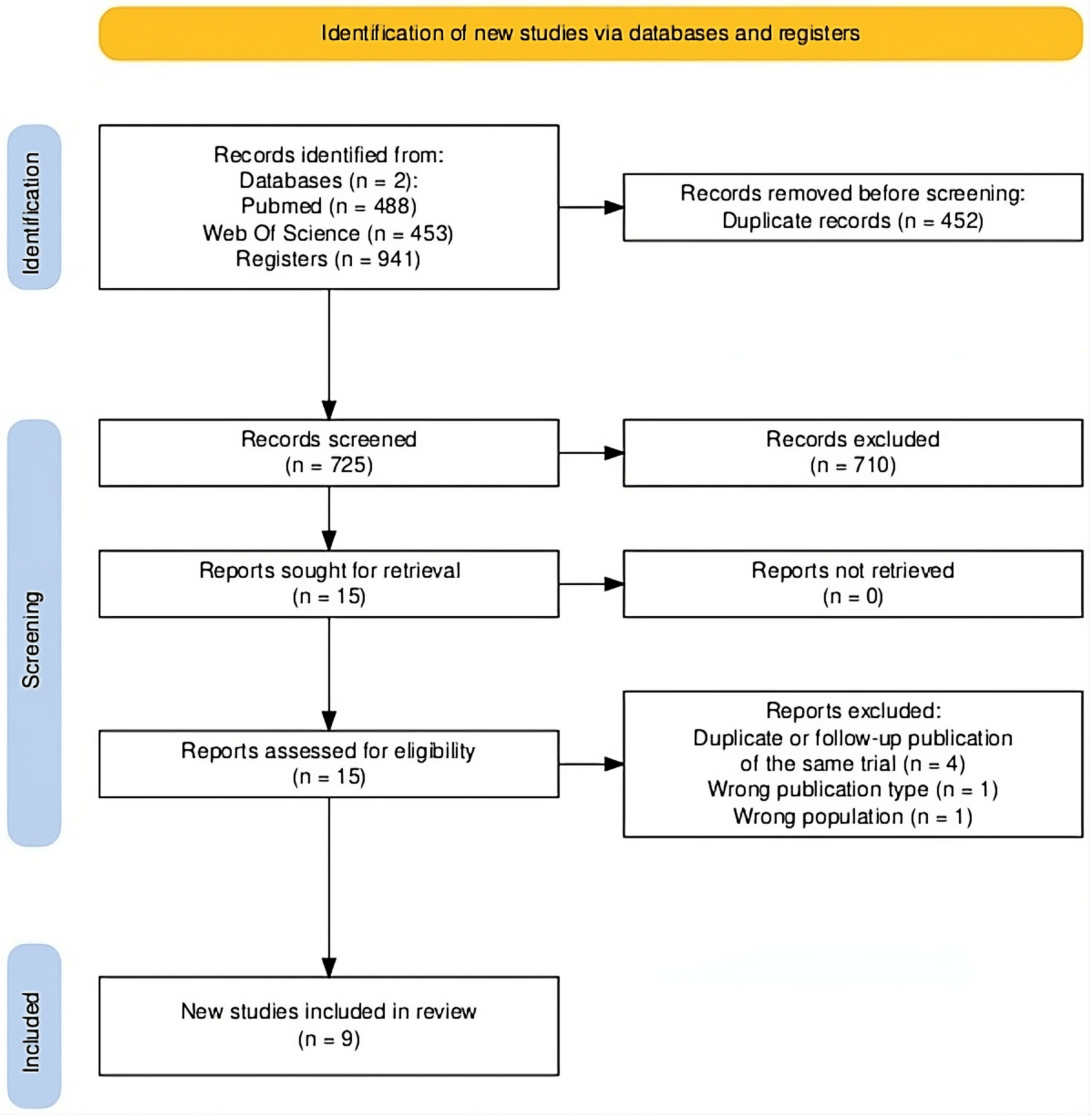
Preferred Reporting Items for Systematic Reviews and Meta-Analyses (PRISMA) diagram for systematic review Records were identified through database searching in MEDLINE (PubMed) and Web of Science (n = 941). Duplicate records were identified and removed using Rayyan software, which applies automated and manual deduplication processes. Consequently, the number of duplicate records does not necessarily correspond to a direct subtraction from the total number of records identified. After deduplication, 725 records were screened, 15 reports were assessed for eligibility, and nine randomized controlled trials were included in the final review.

A total of 725 references were identified from the electronic search of databases and the aforementioned sources. After removing duplicates (n = 452) and initial screening by title and abstract, 710 references were excluded. Therefore, 15 articles were included for comprehensive review; four were excluded because they belonged to the same cohort of clinical trials with more recent publication dates updating the follow-up period [12-15], one article was excluded because the population did not meet the objectives, and finally, another article was excluded because it was not a clinical trial. Finally, nine studies were included for analysis in this systematic review, as shown in the PRISMA flow diagram (Figure 1).

Studies Included

The combined analysis included nine clinical trials involving more than 263,000 subjects, with follow-up periods ranging from five to 26 years. Most populations were in Asia, specifically South Korea and Japan, and only one was in Colombia, regions characterized by high infection prevalence and intermediate to high baseline gastric cancer risk [16-25]. Of these trials, seven recruited healthy community individuals without gastric neoplasia at baseline [17-21,24,25], while one RCT included healthy first-degree relatives of gastric cancer patients [22].

The interventions across these studies included triple, quadruple, or sequential therapies, with control groups receiving placebo, symptomatic management, or no eradication treatment. The detailed characteristics of each trial, diagnostic methods, eradication rates, duration of follow-up, method of cancer confirmation, and risk of bias are summarized in Table 3.

**Table 3:** Characteristics of the included studies [16-25] This table summarizes the main characteristics of the randomized clinical trials included in the systematic review and meta-analysis evaluating *Helicobacter pylori* eradication for the primary prevention of gastric cancer. Information is presented on diagnostic methods for *H. pylori* infection, sample size, population characteristics and study setting, eradication regimens and reported eradication rates, duration of follow-up, methods used for gastric cancer ascertainment, and overall risk of bias assessment.

Study (year)	*Helicobacter pylori* diagnostic method	Sample size (eradication group)	Population and setting	Eradication regimen	Eradication rate	Follow-up duration	Gastric cancer ascertainment	Risk of bias
Piazuelo et al., 2021 [16,25]	Modified Steiner silver stain on gastric biopsies	800 (396)	Adults with gastric precancerous lesions (atrophic gastritis, intestinal metaplasia, dysplasia); high-risk area, Colombia	Triple therapy (omeprazole, amoxicillin, clarithromycin) for 14 days	Not specifically reported; *H. pylori* positivity 97% at baseline, 41% at 20 years	Up to 20 years	Serial endoscopy with multiple biopsies; histological assessment by two blinded GI pathologists; OLGA, OLGIM, and Correa scores	Moderate-Low
Saito et al., 2005 [24]	Not reported	692 (379)	Adults aged 20-59 years; 145 centers, Japan	Lansoprazole 30 mg, amoxicillin 1.5 g, clarithromycin 400 mg once daily for 7 days	74.4%	5 years	Endoscopy with biopsies	Unclear
Wong et al., 2012 [23]	¹³C-urea breath test (¹³C-UBT)	1,024 (510)	Adults aged 35-64 years with *H. pylori* infection and advanced gastric lesions; Linqu County, China	Omeprazole 20 mg, amoxicillin 1 g, clarithromycin 500 mg twice daily for 7 days	PP 78.2%; ITT 71.3%	24 months	Endoscopy with biopsies at five standardized sites; central pathology review	Low
Zhou et al., 2014 [17]	Rapid urease test and Warthin-Starry stain; eradication confirmed by ¹³C-UBT	552 (276)	Adults aged 35-75 years; high-risk rural area, Yantai, Shandong, China	Omeprazole 20 mg, amoxicillin 1 g, clarithromycin 500 mg twice daily for 7 days	88.9% (1 month)	10 years	Surveillance endoscopy with biopsies; Sydney System histology	Moderate-Low
Li et al., 2019 [18]	Baseline IgG serology (ELISA); ¹³C-UBT in subgroup	3,365 (1,130 seropositive)	Adults aged 35-64 years; high-risk region, Linqu County, China	Amoxicillin 1 g + omeprazole 20 mg twice daily for 14 days	Not reported	22.3 years	Scheduled gastroscopies, cancer registry linkage, autopsies, and active follow-up	Low
Choi et al., 2020 [22]	Rapid urease test plus Wright-Giemsa stain (≥2/4 positive)	1,676 (832)	First-degree relatives of gastric cancer patients; 40-65 years; South Korea	Lansoprazole 30 mg, amoxicillin 1,000 mg, clarithromycin 500 mg twice daily for 7 days	70.1% (PP)	Median 9.2 years (max 14.1)	Biennial surveillance endoscopy; confirmation via national cancer registry	Low
Yan et al., 2022 [19]	Rapid urease test plus histology; eradication confirmed by ¹³C-UBT	1,630 (817)	Asymptomatic *H. pylori*-infected adults aged 35-65 years; high-risk area, Fujian, China	Omeprazole, amoxicillin/clavulanate, metronidazole for 14 days; rescue quadruple therapy if failure	83.7%	Up to 26.5 years	Scheduled endoscopies, cancer registry linkage, pathology review, and active follow-up	Low
Lee et al., 2021 [21]	Stool antigen test (HPSA)	152,503 invited (8,664 treated)	Adults aged 50-69 years in a population-based screening program; Changhua County, Taiwan	10-day sequential therapy; levofloxacin-based rescue	91.9% overall; 97.6% in adherent participants	Median 5.7 years (IQR 4.9-6.5)	Taiwan Cancer Registry and Mortality Database	Moderate
Pan et al., 2024 [20]	¹³C-urea breath test (¹³C-UBT)	180,284 (52,026)	Adults aged 25-54 years; high-risk area, Linqu County, Shandong, China	Bismuth-based quadruple therapy for 10 days	72.9% (PP)	11.8 years	Cancer registry linkage, mortality database, and pathological confirmation	Moderate

Reduction in the Incidence of Gastric Cancer

**Figure 2:**
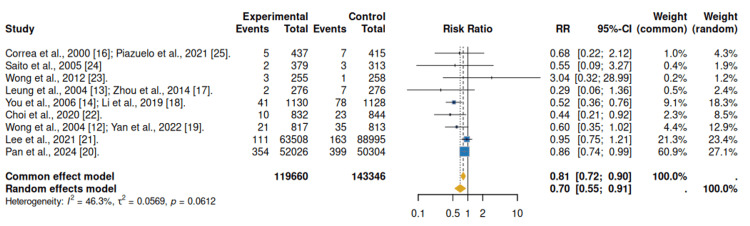
Forest plot: incidence of gastric cancer Forest plot depicting relative risks (RRs) with 95% confidence intervals for gastric cancer incidence comparing *Helicobacter pylori* eradication therapy versus no eradication. Pooled estimates were calculated using fixed- and random-effects models.

Nine clinical studies were selected, all of which were controlled clinical trials that analyzed the effect of *H. pylori *eradication on the incidence of gastric cancer in high-prevalence populations [17-25]. The combined analysis shows a significant reduction in the risk of developing gastric cancer after *H. pylori* eradication. Under a fixed-effect model, the relative risk (RR) was 0.81 (95% CI: 0.72-0.90; p < 0.001), while for the random effects model, RR was 0.70 (95% CI: 0.55-0.91; p = 0.001), with moderate heterogeneity (I² = 46.3%). Therefore, *H. pylori* eradication is associated with a reduction in the risk of gastric cancer of approximately 21-30%, a consistent, clinically relevant, and robust effect (Figure 2).

In terms of clinical relevance, the baseline incidence of gastric cancer in Colombia is between 0.3% and 0.5%, which is consistent with intermediate-risk populations; given that the relative risk (RR) is 0.7, the number needed to treat (NNT) to prevent one case of gastric cancer is estimated to be between 667 and 1,111 people treated over a 10- to 15-year follow-up period (Table 4).

**Table 4:** NNT for the incidence of gastric cancer The table shows the estimated NNT to prevent one case of gastric cancer across different baseline annual incidences (p₀) and relative risk (RR) scenarios. Baseline incidences of 0.3%, 0.5%, and 1.0% were selected to represent low-, intermediate-, and high-risk populations, respectively. NNT, number needed to treat

Baseline incidence (p₀)	NNT (RR = 0.81)	NNT (RR = 0.77, trim-and-fill)	NNT (RR = 0.70)
0.3% (0.003)	1,754	1,449	1,111
0.5% (0.005)	1,053	870	667
1.0% (0.010)	526	435	333

Reduction in Gastric Cancer Mortality

**Figure 3:**
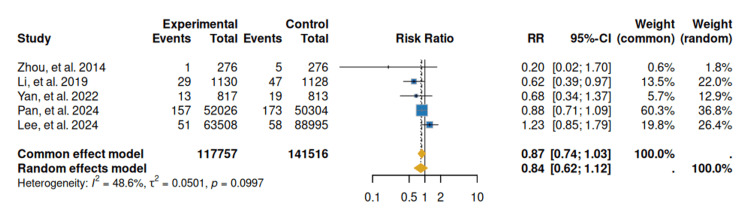
Forest plot: gastric cancer mortality Forest plot depicting risk ratios (RRs) with 95% confidence intervals for gastric cancer–specific mortality comparing *Helicobacter pylori* eradication versus control. References: [17-21]

Regarding the reduction in gastric cancer mortality in patients with *H. pylori* infection, in the combined mortality analysis, neither the fixed-effect nor the random-effect model was statistically significant. However, a reduction in the risk of death was observed, with an RR of 0.87 (95% CI 0.74-1.03) for the fixed-effects model, while the random-effects model yielded an RR of 0.84 (95% CI 0.62-1.12; p = 0.0997) [17-21], with moderate heterogeneity (I² = 48.6%). Therefore, the evidence is insufficient for a definitive conclusion (Figure 3).

Assessment of Risk of Bias in Included Studies

In this systematic review of the selected studies, a rigorous assessment of the risks of bias associated with each study was carried out using the RoB 2.0 tool. Based on a review of five key dimensions: D1. The randomization process, D2. Deviations from the planned interventions, D3. Missing outcome data, D4. Outcome measurement, and finally D5. Selective reporting of results.

Most of the studies included in the combined analysis described adequate randomization procedures and maintained acceptable adherence to interventions, with no evidence of systematic deviations that would compromise internal validity. Regarding the management of incomplete outcome data (D3), there was one case where the magnitude and pattern of missing data were considered significant enough to classify this domain as high risk, which directly affected the overall assessment of the study [25].

The measurement of outcome assessment bias was low, as the studies used histopathological verification or standardized national registries for the identification of gastric cancer, reducing the possibility of classification errors or differential measurements between groups.

**Figure 4:**
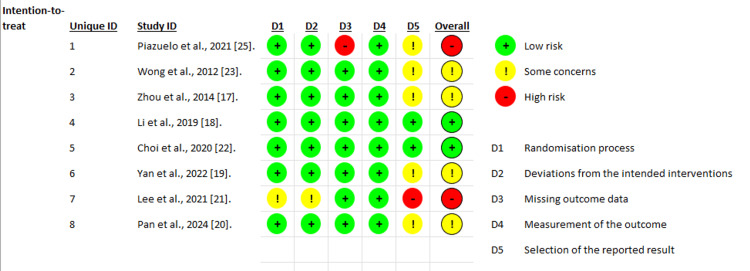
Summary of risk of bias (RoB 2.0) Summary of the risk of bias assessment for the included randomized clinical trials using the Cochrane Risk of Bias 2.0 (RoB 2.0) tool. Each domain is classified as low risk, some concerns, or high risk of bias, providing an overall judgment of methodological quality across studies.

In the domain of outcome selection, several trials identified the availability of multiple definitions, time points, or potentially eligible analyses to measure the outcome, without the existence of a pre-published protocol confirming the pre-specification of the analyses. This led to several studies being classified as “some concerns” and contributed to the overall assessment of intermediate risk in these studies (Figure 4).

Finally, one study was only available as a conference abstract, which prevents a formal evaluation of its methodological domains; therefore, it was classified as not assessable. Overall, although most trials had a low to moderate risk of bias, limitations remain, mainly related to outcome reporting and missing data management, which should be considered when interpreting the certainty and robustness of the evidence analyzed.

Assessment of publication bias

Publication Bias for Gastric Cancer Incidence

**Figure 5:**
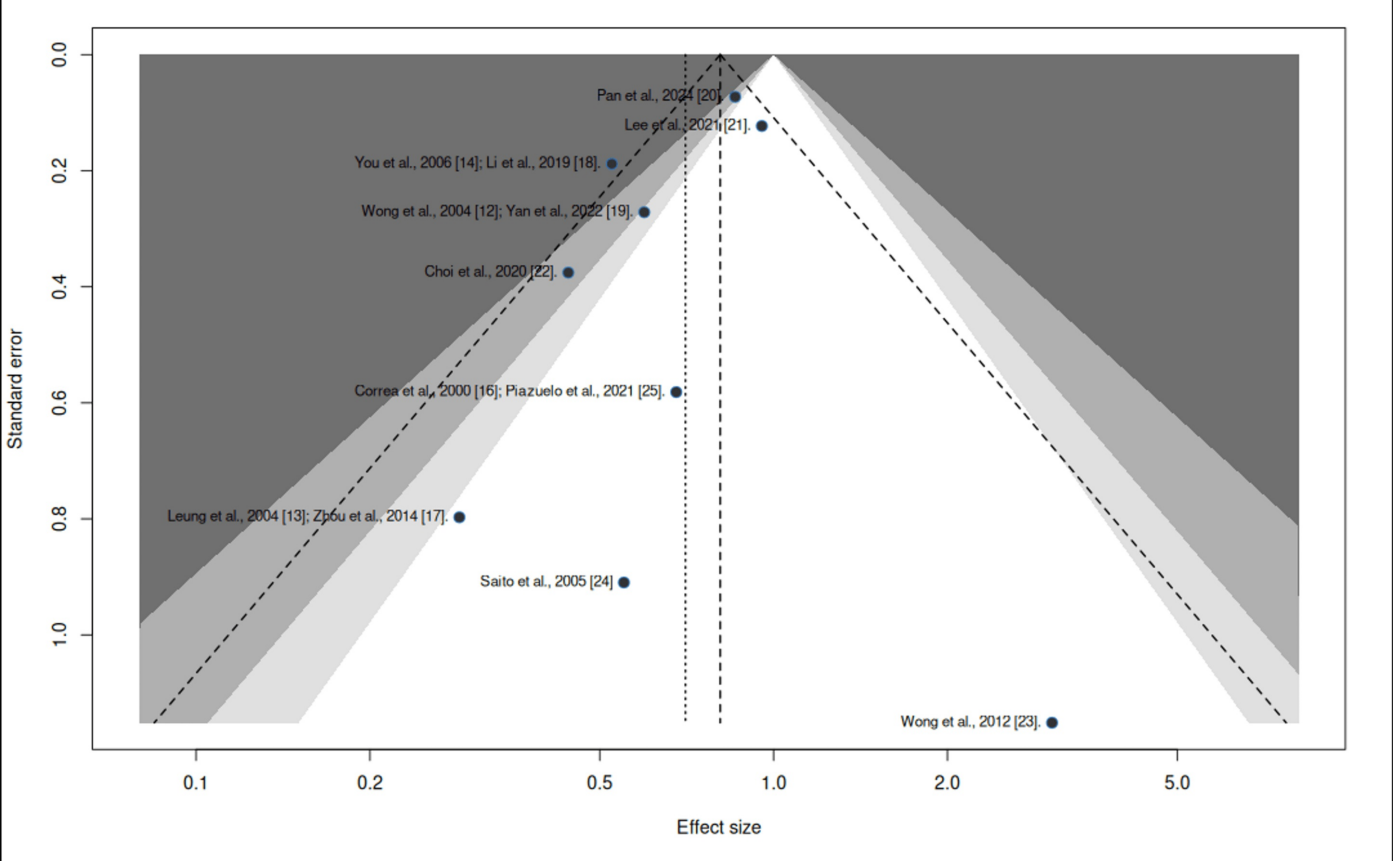
Funnel plot: publication bias Funnel plot of included studies assessing potential publication bias in gastric cancer incidence outcomes.

To assess publication bias, we used a funnel plot, which showed evidence of asymmetry in the distribution, with a tendency to concentrate toward the left side of the graph (Figure 5). This suggests possible publication bias. Therefore, due to the limited number of clinical trials (<10) and the moderate heterogeneity observed, the Egger test was not applied, as its statistical viability is insufficient in this scenario and may provide false negatives.

**Figure 6:**
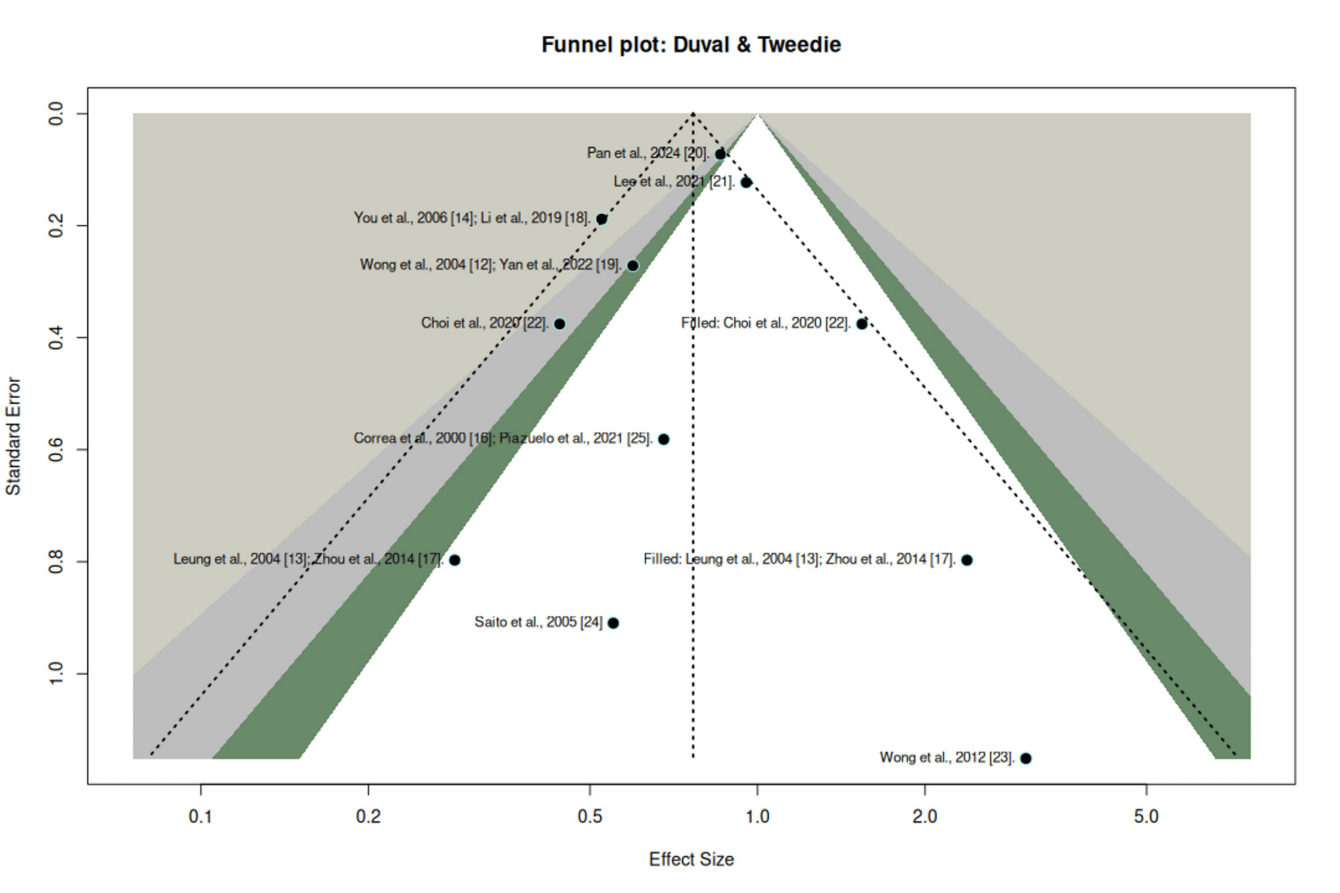
Funnel plot: sensitivity analysis for publication bias using an imputation-based approach Sensitivity analysis evaluating the robustness of the pooled effect estimate after accounting for potential publication bias using an imputation-based method. The adjusted pooled estimate suggests that the overall effect remains consistent after correction.

To assess the robustness of the findings in the presence of potential publication bias, a sensitivity analysis based on funnel plot asymmetry adjustment was performed. After imputing two potentially missing studies, the pooled estimate remained statistically significant, yielding an adjusted RR of 0.76 (95% CI: 0.59-0.98; p = 0.0396), with moderate heterogeneity (I² = 48.9%). These results indicate that the observed protective effect persists despite potential small-study effects and does not materially affect the overall conclusions (Figure 6).

Discussion

This meta-analysis is based on more than 250,000 subjects with prolonged follow-up. It confirms that *H. pylori* eradication significantly reduces the incidence of gastric cancer, with a clinically relevant effect size (RR = 0.70), under moderate heterogeneity (I² = 46.3%). In other words, eradication of *H. pylori* in asymptomatic patients reduces gastric cancer by up to 30% in high-risk populations. These findings underscore the importance of *H. pylori* as a primary causative agent in the sequence leading to adenocarcinoma. Eradication, when performed before irreversible changes in the gastric mucosa, is substantial for prevention.

These findings are in line with those reported in previous meta-analyses [7,26,27]. Methodological transparency was strengthened by incorporating a sensitivity analysis to account for the potential influence of publication bias. After applying a conservative adjustment approach, the overall effect estimates remained robust, supporting the consistency of the results.

This provides solid stability of the combined effect, showing that even after imputing missing studies, the protective effect remains (adjusted RR = 0.76; 95% CI: 0.59-0.98). This result reaffirms the association and suggests that *H. pylori* eradication reduces the risk of gastric cancer regardless of possible publication biases. Therefore, the present meta-analysis provides a relevant methodological approach, reporting the findings with adequate credibility and allowing for a better assessment of applicability in the public health context.

The analysis of gastric cancer-specific mortality, which included five trials of high methodological quality, showed a favorable trend but did not reach statistical significance (RR = 0.84; 95% CI: 0.62-1.12). In contrast, this result partially differs from a recently published meta-analysis [28], which reported a significant effect. A possible explanation for this discrepancy lies in methodological differences during data extraction, particularly in large studies such as that of Pan et al. [20], whose trial presents multiple analyses (by ITT, by protocol, and by infection status strata) that must be carefully considered when consolidating mortality events. In our analysis, we used global ITT data, which represent the most conservative estimate in accordance with methodological guidelines.

However, the directional consistency of the results suggests a real clinical benefit, particularly in studies with longer follow-up and adherence to eradication treatment. None of the trials reported an increase in overall mortality, confirming the long-term safety of the antibiotic therapies used.

The heterogeneity observed in the estimated effects can be attributed to differences between studies in terms of design, baseline characteristics of participants, type of antibiotic regimen, and duration of follow-up. However, the direction of the effect was consistent, and the magnitude of the benefit remained stable even in sensitivity analyses, strengthening the validity of the conclusions.

Another element to consider is the NNT obtained, which differs in magnitude from previous meta-analyses, perhaps explained by methodological differences and the consideration of the baseline risk with which it was calculated, which will clearly vary considering the population risk, such as in Asian populations, where the risk is high, or Latin American populations, where the risk is intermediate. Nevertheless, the estimated NNT is within the threshold generally accepted as clinically useful in cancer prevention, particularly when the intervention is safe, low-cost, and targeted at a high-mortality neoplasm. This level of impact is comparable to that observed in widely adopted preventive strategies for other cancers [29].

In this context, pragmatic multicenter trials are needed to evaluate the effectiveness and cost-effectiveness of systematic eradication under real-world clinical practice conditions, incorporating local variables such as the prevalence of virulent strains, resistance patterns, and reinfection rates. However, the results of this meta-analysis suggest that, even under conservative scenarios, *H. pylori* eradication represents a cost-effective intervention in intermediate-risk populations, particularly when the costs are covered by policies, supplemental plans, or prepaid health insurance. In these settings, the removal of economic barriers increases the viability and sustainability of screening, making it a rational primary cancer prevention measure.

Limitations

This meta-analysis has several limitations that should be considered when interpreting the results. First, the included trials showed substantial methodological heterogeneity, derived from essential methodological differences between the included trials, both in design (individual clinical trials versus cluster community interventions) and in the duration of follow-up and therapeutic regimens used for *H. pylori* eradication. These variations may introduce non-random heterogeneity and limit the direct comparability of results.

Second, the definition of the primary outcome (incidence of gastric cancer) varied between studies: some used histological confirmation, while others relied on population registries or clinical diagnosis. In addition, stratification by histological type (cardial vs. non-cardial) or by severity of preneoplastic lesion was not uniform, which could have diluted the actual effect of treatment in specific risk subgroups.

A third limitation is that few studies reported specific mortality from gastric cancer, and in several of them, this outcome corresponded to a rare event, with limited statistical power to detect significant differences. This explains why the effect on mortality, although directionally favorable, did not reach statistical significance in the overall meta-analysis.

Another important limitation is the geographical homogeneity of the included studies. Of all the evidence evaluated, only one study was conducted outside Asia, a region where the incidence and mortality of gastric cancer are the highest worldwide, which could lead to an overestimation of the effect of eradication in populations with a lower baseline risk. Although Latin America is the second most affected region globally, its representation in our analysis is incomplete, as the only non-Asian study evaluates a population from a high-risk area within the region. Therefore, significant questions arise about the generalizability of the protective effect of the intervention to other geographic populations and those with a more moderate risk profile.

From a statistical perspective, formal regression-based tests for small-study effects were not applied because the number of included trials was below the recommended threshold for reliable implementation. Instead, an adjustment based on funnel plot asymmetry was used as a complementary sensitivity analysis to explore more conservative scenarios. This approach strengthens the interpretability of the findings by assessing the robustness of the pooled estimates. Nevertheless, it should be acknowledged that the reliability of publication bias assessments is inherently limited when fewer than 10-15 studies are available, as in the present meta-analysis.

Although the evidence consolidated in this meta-analysis supports the efficacy of *H. pylori* eradication as a primary prevention strategy for gastric cancer, structural factors that limit its universal application persist. The lack of homogeneous data on long-term reinfection, therapeutic adherence, and antibiotic resistance prevents the sustained magnitude of the benefit in public health programs from being established with certainty. These elements are especially relevant in regions where treatment coverage and endoscopic follow-up are uneven, as is the case in much of Latin America.

These results support the efficacy of *H. pylori* eradication as a primary preventive strategy against gastric cancer, especially in intermediate-risk settings, where the prevalence of infection exceeds 70%, and gastric cancer mortality rates are high [30].

In summary, the systematic eradication of *H. pylori* should be considered an ethical, clinically justifiable, and economically viable strategy to reduce the future burden of gastric cancer, provided that it is integrated into controlled programs and accompanied by local antibiotic surveillance and health education policies.

## Conclusions

Our meta-analysis indicates that *H. pylori* eradication is associated with a reduced risk of gastric cancer and suggests a sustained protective effect over long-term follow-up. Despite substantial between-study heterogeneity and some funnel plot asymmetry, we found no statistically significant evidence of publication bias. Overall, the findings indicate a net protective benefit of the intervention and may inform the development of population-based screening and eradication programs in intermediate- and high-risk settings, including regions with epidemiological profiles similar to that of Colombia. In health systems with sufficient coverage, *H. pylori* screening and eradication may represent a feasible component of primary gastric cancer prevention; however, implementation should consider local epidemiology, resource availability, and formal cost-effectiveness assessments.
